# Does DRD2 Taq1A Mediate Aripiprazole-Induced Gambling Disorder? A Pharmacogenetic Hypothesis

**DOI:** 10.3389/fpsyt.2020.00275

**Published:** 2020-04-15

**Authors:** Andrea Miuli, Mauro Pettorruso, Ester Romanelli, Gianfranco Stigliano, Daniela Di Giuda, Fabio De-Giorgio, Giovanni Martinotti, Massimo di Giannantonio

**Affiliations:** ^1^ Department of Neuroscience, Imaging and Clinical Sciences, “G. d'Annunzio” University, Chieti, Italy; ^2^ Medical Genetics Unit, San Pietro Fatebenefratelli Hospital, Rome, Italy; ^3^ Institute of Nuclear Medicine, Fondazione Policlinico Universitario “A. Gemelli”, Università Cattolica del Sacro Cuore, Rome, Italy; ^4^ Section of Legal Medicine, Institute of Public Health, Università Cattolica del Sacro Cuore, Rome, Italy; ^5^ Department of Pharmacy, Pharmacology, Clinical Science, University of Hertfordshire, Hatfield, United Kingdom

**Keywords:** aripiprazole, gambling disorder, pharmacogenetic, DRD2Taq1A, polymorphism, ARI-induced GD

## Abstract

Second-generation antipsychotics (SGA) are a pharmacological class widely used in psychiatry thanks to their efficacy and good tolerability profile. One of the most used SGA is aripiprazole (ARI) because of its several formulations and safe metabolic and cardiac profile. As reported in a recent review, there are growing numbers of reports about ARI-induced gambling disorder (ARI-induced GD) which should encourage clinicians to use ARI more cautiously. Given the common genetic susceptibility of both GD and ARI's clinical response to a genetic polymorphism on the D2 receptor (DRD2/ANKK1 Taq1A; rs1800497), the hypothesis regarding the origin of this phenomenon could be found in the altered sensitization of dopamine's receptors that certain individuals carry genetically. The identification of a possible genetic susceptibility (detectable by genetic tests) could provide clinicians with an explanation for the ARI-induced GD and the possibility of using genetic screening tools for those cases of suspected predisposition; this would allow the clinician to prescribe ARI with less apprehension. The confirmation of this hypothesis through future pharmacogenetic studies may be useful for clinicians to have a correct understanding of the phenomenon.

## Introduction

Second-generation antipsychotics (SGA) have been widely used in psychiatry, both as a first-line treatment in psychosis (in some cases even more than first-generation antipsychotics) and in several other psychiatric diseases. Their widespread diffusion has been possible not only because of their good efficacy, but also for the low amount of side effects resulting from the therapy (1). Nowadays, they are used not only for the treatment of schizophrenia but also as mood stabilizers, sedatives for psychomotor agitation, and for the treatment of behavioral disorders (2). Currently, one of the most commonly used SGA in psychiatry is aripiprazole (ARI). At a pharmacological level ARI, as well as brexpiprazole and cariprazine, is a partial D2 receptor agonist, and it shows a very high affinity for 5-HT1A (5-HT1AR), 5-HT2A (5-HT2AR), 5-HT2B (5-HT2BR), and 5-HT7 (5-HT7R) receptors (3). ARI is also a partial agonist of D3 receptors. These receptors, in particular, strongly present at mesolimbic level, could explain the problems of impulse dysregulation related to treatment with ARI or dopamine agonists (pramipexole and ropinirole) (4). Its widespread use is given not only by a large number of existing formulations (capsules with various dosages, solution, vials, depot) but also because of its good safety profile (especially in terms of cardiac safety) (5).

### Aripiprazole-Induced Gambling Disorder: Recent Evidence

Nowadays, one of the most studied behavioral addictions is gambling disorder (GD) (6, 7). Although GD neurobiological underpinnings are largely unknown, alterations in dopaminergic pathways have been extensively hypothesized (6–8). Currently there is an increasing interest in ARI-induced GD risk reports as shown in a recent review in 2019 (9) which collects 16 case reports of GD, developed after the consumption of an ARI therapy for schizophrenia. It is interesting to note that, in another recent review on ARI-induced GD, most of the selected cases (16 out of 23) show a complete remission of the GD-like symptomatology after the discontinuation of ARI therapy and in two after the daily dosage reduction, giving a useful reflection on the onset and possible management of ARI-induced GD (10). The explanation for the ARI-induced GD may be found in upregulation of dopaminergic receptors, which may cause a higher sensitivity to ARI. The relation between the full dopamine agonist used, for example, in Parkinson's disease (e.g., pramipexole and ropinirole) and GD-like behaviors is well demonstrated, but the occurrence of GD after partial agonist treatment requires specific assessments and randomized trials with a higher number of participants (9). The reason behind this difficult detection of ARI-related GD may be due to this adverse event occurring within a small population of ARI users with particular drug response patterns.

### DRD2 Polymorphism and Aripiprazole-Induced Gambling Disorder

A possible explanation for this could be found in the genetic heritage of these individuals, in particular in DRD2 polymorphism. One of the best studied polymorphisms, whose frequency in the world population is 33% (11), is the DRD2/ANKK1 Taq1A polymorphism (rs1800497), located on chromosome 11q23.2 and associated with a reduced number of dopamine binding sites in the brain (12). Increasing evidence shows this polymorphism (DRD2*A1) in particular, having a strong relationship to some neurological and psychiatric conditions. On a neurological level, DRD2*A1 seems related to a constriction of caudate volume in elderly subjects without dementia (13) and also to a worsening in memory performance, especially during long-term memory update (14). On a psychiatric level, this DRD2Taq1A polymorphism is associated with depression and addiction behaviors (15). This genotype is involved not only in the process of development of addiction, but also in treatment response; in particular, DRD2*A1 has an important role in the process of abstinence from smoking (16), it can predict a good response when disulfiram is used for the treatment of cocaine addiction (17) and it has also been related with a higher probability of fatal methamphetamine intoxication (18). DRD2 A1/A1 genotype appears associated with the development of an antisocial personality disorder (19) as well and with psychopathic-like behaviors in alcohol-dependent patients (20). Although DRD2Taq1A polymorphism does not seem to be linked to Internet Gaming Disorder (21), some evidence suggests its possible correlation with GD. While studying impulsivity traits Comings and coll., in 1997, showed that there was a higher percentage of subjects with DRD2 A1/A2 genetic variables in the GD group than in the Tourette Syndrome or smokers' group (22). Regarding cognitive domains, Fagundo and colleagues in 2014 reported a significant predisposition to lower cognitive levels in carriers of DRD2Taq1A polymorphism (23).

In addition to modulating the pharmacological response in the treatment of addictions, DRD2Taq1A polymorphism seems to mediate the effects of SGA [inducing akathisia more frequently (24) and increasing prolactin levels activated by antipsychotics (25)] and, mostly, the effects of ARI. In 2008 this association was studied among healthy controls, revealing that ARI decreased the metabolism of subjects with A2/A2 genotype in some sections of their frontal and temporal lobe, in the right cingulate gyrus. According to the authors, this modulation of metabolism could change the effect of ARI even in pathological samples (26). Again in 2008, researchers investigated the ARI response on 90 schizophrenic patients, considering all DRD2Taq1A polymorphisms (14 patients were A1/A1, 51 patients were A1/A2, and 25 patients were A2/A2). This work, for the first time, provided evidence that patients carrying the A1/A1 genotype had a better therapeutic response to ARI (27). In addition, DRD2*A1 seems to be related with higher levels of plasma homovanillic acid during the treatment of schizophrenia with ARI, and this could be related to a decrease in dopaminergic neuronal firing in the brain (28). Unlike ARI, there is currently no scientific evidence of correlation in the literature between the DRD2Taq1A polymorphism and other partial D2 agonists (such as brexiprazole and cariprazine), while the lower impulse control given by dopamine agonist therapy (pramipexole and ropinirole) is well known in subjects with this polymorphism (29).

## Discussion

Given the association between DRD2 polymorphism and the development of adverse events related to dopaminergic sensibilization in SGA treatment, together with the different metabolism in the frontal regions of healthy subjects' brains, as well as the different responses of schizophrenic patients treated with ARI, one of the reasons why ARI-induced GD could develop, could be found in genes that influence the dopaminergic pathways.

Since GD is a very disabling behavior, with burdening economic consequences and severe social harm for the patient (30), the identification of an endophenotype that can lead a person to become genetically sensitive to the development of ARI-induced GD could not only prevent the occurrence of this side effect, but also clarify the diagnostic doubt around its diagnosis. Eventually, since ARI has a very good safety profile, identifying such predictive genetic markers can improve clinicians' perception of its safety. There would also be several medical-legal advantages related to this.

We therefore hope that, based on the hypothesis of this paper, there will be future pharmacogenetic studies to investigate the direct association of ARI-induced GD, including the concept of endophenotype, also in the context of adverse events in ARI pharmacotherapies.

## Author Contributions 

AM, MP, GM, and MG conceptualized the hypothesis. AM, GS, and ER wrote the first draft of the manuscript. MP, FD-G, DG, GM, and MG reviewed the first draft. All authors have contributed to, and have approved, the final manuscript.

## Funding

This work was supported by the “Departments of Excellence 2018-2022” initiative of the Italian Ministry of Education, University and Research for the Department of Neuroscience, Imaging and Clinical Sciences (DNISC) of the University of Chieti-Pescara.

## Conflict of Interest

The authors declare that the research was conducted in the absence of any commercial or financial relationships that could be construed as a potential conflict of interest.
